# Draft Genome Sequences of Escherichia coli Strains FP2 and FP3, Isolated from the Canada Goose (Branta canadensis)

**DOI:** 10.1128/MRA.01079-18

**Published:** 2018-09-06

**Authors:** Allison L. Denny, Susan E. Arruda

**Affiliations:** a Department of Biology, Franklin Pierce University, Rindge, New Hampshire, USA; Queens College

## Abstract

Draft genomes of two strains of Escherichia coli, FP2 and FP3, isolated from the feces of the Canada goose (Branta canadensis), were sequenced. Genome sizes were 5.26 Mb with a predicted G+C content of 50.54% (FP2) and 5.07 Mb with a predicted G+C content of 50.41% (FP3).

## ANNOUNCEMENT

Escherichia coli bacteria are rod-shaped, Gram-negative, facultative aerobes that are often encapsulated (1–3). The E. coli genome consists of a single circular chromosome that ranges from 4.5 to 5.9 million base pairs and comprises 4,000 to 5,500 genes (2, 4, 5). Like some other members of the Enterobacteriaceae family (e.g., Serratia marcescens), E. coli bacteria have pathogenic and nonpathogenic strains (6). Pathogenic strains can be categorized into different pathotypes based on which strategy is used to interact with the host cell and the degree of virulence (2, 7). Various strains of nonpathogenic E. coli are known to be the principal inhabitants of mammalian gut microbiomes and have also been found in the intestinal tract of birds, reptiles, and fish (2, 8).

E. coli strains were obtained as environmental isolates on the Franklin Pierce University campus in Rindge, New Hampshire. Samples of Canada goose feces were streaked onto lysogeny broth agar and incubated overnight at 37°C. Single colonies were used to inoculate lysogeny broth from which genomic DNA was isolated with the QIAamp DNA purification minikit (Qiagen, Bethesda, MD, USA). Fragmented genomic DNA was tagged with adapters using the KAPA HyperPlus kit (Wilmington, MA, USA) and then loaded on an Illumina HiSeq 2500 instrument by the Hubbard Center for Genome Studies (University of New Hampshire, Durham, NH, USA) for sequencing (FP2, 8,418,266 reads, 188× coverage; FP3, 8,418,266 reads, 225× coverage). The 250-bp paired-end reads were trimmed using Trimmomatic version 3.5 (default settings), and genome sequences underwent *de novo* assembly using SPAdes version 3.9.0 (using default settings) (9, 10). Contigs less than 500 bp were removed before the genome was submitted for annotation (2 July 2018) with the NCBI Prokaryotic Genome Annotation Pipeline (PGAP) (11).

The FP2 genome is approximately 5,257,000 bp, distributed in 274 contigs (the largest is 330,378 bp), with an overall G+C content of 50.54% and an *N*_50_ of 148,984 bp. PGAP predicted 5,127 protein-coding genes, 21 rRNA genes, 82 tRNA genes, and 245 pseudogenes. The FP3 genome is approximately 5,068,010 bp, distributed in 297 contigs (the largest is 721,880 bp), with an overall G+C content of 50.41% and an *N*_50_ of 600,909 bp. PGAP identified 4,836 protein-coding genes, 13 rRNA genes, 84 tRNA genes, and 261 pseudogenes. The similarity between these genomes and other known E. coli genomes (e.g., GenBank accession numbers CP022393 and LT883142), along with the manner in which the samples were isolated, suggests that FP2 and FP3 are likely residents of the Branta canadensis gut microbiome.

### Data availability.

The FP2 and FP3 whole-genome shotgun sequences were deposited in DDBJ/ENA/GenBank under the accession numbers QNRA00000000 and QNRB00000000, respectively. The versions described in this paper are versions QNRA01000000 and QNRB01000000, respectively.
